# Orally desensitized mast cells form a regulatory network with Treg cells for the control of food allergy

**DOI:** 10.1038/s41385-020-00358-3

**Published:** 2020-12-10

**Authors:** Yoshihiro Takasato, Yosuke Kurashima, Masahiro Kiuchi, Kiyoshi Hirahara, Sayuri Murasaki, Fujimi Arai, Kumi Izawa, Ayako Kaitani, Kaoru Shimada, Yukari Saito, Shota Toyoshima, Miho Nakamura, Kumiko Fujisawa, Yoshimichi Okayama, Jun Kunisawa, Masato Kubo, Naoki Takemura, Satoshi Uematsu, Shizuo Akira, Jiro Kitaura, Takao Takahashi, Toshinori Nakayama, Hiroshi Kiyono

**Affiliations:** 1 Department of Mucosal Immunology, The University of Tokyo Distinguished Professor Unit, The Institute of Medical Science, The University of Tokyo, Tokyo, 108-8639 Japan; 2 Department of Pediatrics, Keio University School of Medicine, Tokyo, 160-8582 Japan; 3 Department of Innovative Medicine and Mucosal Immunology, Graduate School of Medicine, Chiba University, Chiba, 260-8670 Japan; 4 International Research and Development Center for Mucosal Vaccines, The Institute of Medical Science, The University of Tokyo, Tokyo, 108–8639 Japan; 5 Division of Gastroenterology, Department of Medicine, CU-UCSD Center for Mucosal Immunology, Allergy and Vaccines (CU-UCSD cMAV), University of California, San Diego, CA 92093-0956 USA; 6 Institute for Global Prominent Research, Chiba University, Chiba, 260-8670 Japan; 7 Laboratory of Vaccine Materials, Center for Vaccine and Adjuvant Research and Laboratory of Gut Environmental System, National Institutes of Biomedical Innovation, Health and Nutrition, Osaka, 567-0085 Japan; 8 Department of Immunology, Graduate School of Medicine, Chiba University, Chiba, 260-8670 Japan; 9 Atopy Research Center, Juntendo University Graduate School of Medicine, Tokyo, 113-8421 Japan; 10 Allergy and Immunology Research Project Team, Research Institute of Medical Science, Center for Allergy, Center for Medical Education, Nihon University School of Medicine, Tokyo, 173-8610 Japan; 11 Laboratory for Cytokine Regulation, RIKEN Center for Integrative Medical Sciences, Yokohama, Kanagawa 230-0045 Japan; 12 Division of Molecular Pathology, Research Institute for Biomedical Science, Tokyo University of Science, Chiba, 278-0022 Japan; 13 Laboratory of Bioresponse Regulation, Graduate School of Pharmaceutical Sciences, Osaka University, 1-6 Yamada-oka, Suita, Osaka, 565-0871 Japan; 14 Department of Immunology and Genomics, Osaka City University Graduate School of Medicine, 1-4-3 Asahi-machi, Abeno-ku, Osaka, 545-8585 Japan; 15 Laboratory of Host Defense, WPI Immunology Frontier Research Center, Osaka University, Osaka, 565-0871 Japan; 16 Department of Host Defense, Research Institute for Microbial Diseases, Osaka University, Osaka, 565-0871 Japan

## Abstract

Oral immunotherapy (OIT) is an effective approach to controlling food allergy. Although the detailed molecular and cellular mechanisms of OIT are unknown currently, they must be understood to advance the treatment of allergic diseases in general. To elucidate the mechanisms of OIT, especially during the immunological transition from desensitization to allergy regulation, we generated a clinical OIT murine model and used it to examine immunological events of OIT. We found that in mice that completed OIT successfully, desensitized mast cells (MCs) showed functionally beneficial alterations, such as increased induction of regulatory cytokines and enhanced expansion of regulatory T cells. Importantly, these regulatory-T-cell-mediated inhibitions of allergic responses were dramatically decreased in mice lacking OIT-induced desensitized MC. Collectively, these findings show that the desensitization process modulates the activation of MCs, leading directly to enhanced induction of regulatory-T-cell expansion and promotion of clinical allergic unresponsiveness. Our results suggest that efficiently inducing regulatory MCs is a novel strategy for the treatment of allergic disease.

## Introduction

The numbers of patients with allergic diseases have increased worldwide, and about 30% of adults and about 50% of infants now experience allergic diseases such as hay fever and food allergy.^1,2^ About 5% of children have severe food allergy; the lack of curative treatments means that these children require intensive management to avoid intake of food allergens.^1,2^ The clinical signs of food allergic reaction are vomiting, diarrhea, and occasionally life-threatening anaphylaxis.^1,2^ IgE-mediated anaphylaxis is associated with gastrointestinal symptoms, including watery diarrhea, in 25–30% of cases.^3,4^

The central and pathological pathways of those allergic signs are mediated by mast cells (MC)^5^ and their derived mediators, including histamine, serotonin, sphingolipids, and leukotrienes, after MC degranulation induced through the cell surface complexing of FcεR and antigen- specific IgE.^6^ Increased numbers of MCs in the gastrointestinal tract and their activation increases intestinal epithelial permeability, leading to the loss of electrolytes and water (diarrhea) and increasing vasopermeability; these factors potentially can cause systemic anaphylaxis.^7^ Likewise, systemic mastocytosis with gastrointestinal symptoms (e.g., watery allergic diarrhea) increases the risk of severe anaphylaxis.^8^

Inhibition of MC degranulation or blockade of the corresponding receptors of MC-derived mediators (e.g., histamine and leukotrienes) is a widely accepted symptomatic therapy.^9^ In addition, Th2 cytokines produced by MCs, such as IL-4 and IL-5, augment the Th2 response.^10^ IL-4 release increases Th2 cell induction simultaneously with IgE production.^10^ Therefore, degranulation of, and pathogenic IL-4 production by, MCs are essential targets for the treatment of allergic diseases. Accumulation of MCs in the local mucosa, such as that of the gastrointestinal tract and colon, is often observed during food-antigen-induced allergic diarrhea.^11,12^ Inhibition of MC accumulation at the local mucosa is an attractive strategy for regulating the allergic reactions associated with food antigens.^11,12^

Allergen-specific immunotherapy—especially subcutaneous or sublingual administration of allergens—effectively reduces allergic reactions in atopic dermatitis and rhinitis.^13^ Allergen desensitization via the oral route—oral immunotherapy (OIT)—is considered as an effective way of controlling food allergy.^14,15^ However, the underlying cellular and molecular mechanisms of OIT are still lacking in terms of long-term efficacy and safety; thus, elucidation of detailed OIT-mediated immunological events is required to develop and improve OIT-based fundamental treatment of allergic diseases. In addition, most published mechanisms have been based on peripheral blood studies that have analyzed responsiveness to allergens by using markers of degranulation (e.g., CD203) of basophils,^16^ and limited information is available regarding the role of gut mucosa and its associated mucosal immune system.

OIT consists of an initial escalation phase (or acute desensitization), followed by a maintenance (or consolidation) phase.^16,17^ Successful desensitization of MCs by continuous treatment with an allergen is essential for limiting the allergic reaction and leads to the establishment of allergen unresponsiveness (tolerance).^18^ The OIT protocol that was initially proposed and adopted was to increase the threshold of reactivity to the allergen by stimulation with a subthreshold dose, gradually escalating the amount given.^18^ However, the precise mechanisms of immunological transition especially from the initial phase of OIT to the maintenance or consolidation phase to induce unresponsiveness have not been carefully elucidated. Comparison of the characteristics of local MCs in the allergic state and in OIT is required for us to understand the mechanisms of the OIT-induced desensitized condition and to evaluate the efficiency of allergy control by OIT. Previous studies have revealed the novel functions of MCs that acquire immunomodulatory roles by producing regulatory cytokines (e.g., IL-2, IL-10).^19,20^ However, there are technical and ethical difficulties in studying cellular mechanisms in human subjects about mucosal tissues. To overcome these problems, several studies have used an OIT mouse model that might provide important new insights into OIT effects in local tissues (e.g., the intestinal tract), focusing on modulation of the functions of effector cells, especially MCs. Therefore, elucidation of detailed mechanisms of OIT-mediated desensitization system is required for fundamental treatment of allergic diseases.

Here, to reveal the contribution and functions of MCs in immunological transition from allergic promoter to suppressor during the initial phase of OIT in the gut mucosal compartment, we established an OIT murine model that mimicked clinical OIT to characterize the intestinal desensitized-MC-mediated suppression of food allergy. We show the immunoregulatory roles of desensitized regulatory MCs upon OIT in promoting clinical unresponsiveness by the direct induction of regulatory-T-cell (Treg) population expansion. We have uncovered novel roles for orally desensitized regulatory MCs in their involvement in long-lasting oral unresponsiveness.

## Results

### Development of OIT model for control of allergic diarrhea

Food allergy is a type I allergic reaction induced by MC activation.^17^ Increased numbers of MCs and their activation in the mucosal compartment of the intestine enhance gastrointestinal permeability, causing watery diarrhea and allergen dissemination to the systemic compartment, where the allergens become triggers for anaphylaxis.^4^ To evaluate the immunological machinery of intestinal MCs in food allergy, we sought to generate OVA-induced allergic diarrhea in two murine models of MC deficiency, namely BALB/c back-crossed *Kit*^*W-sh/W-sh*^ mice and Mas-TRECK mice; the Mas-TRECK model was derived through continuous administration of diphtheria toxin.^21,22^ Oral challenge with OVA failed to elicit signs of allergic diarrhea in either strain of MC-deficient mice, whereas identically challenged wild-type mice developed severe allergic diarrhea (Supplementary Fig. 1 a–c). When we discontinued the administration of diphtheria toxin for MC depletion to Mas-TRECK mice, the mice started showing allergic diarrhea after oral challenge with OVA (Supplementary Fig. 1c). In addition, anti-IgE treatment suppressed allergic diarrhea (data not shown). This series of experiments directly indicated the indispensable roles of IgE-mediated MC activations in the development of allergic diarrhea.

Next, to establish an optimal OIT protocol for the control of pathological and clinical intestinal signs of food allergy in wild-type Balb/c mice, we tested a OIT protocol involving dose escalation of heated OVA (maximum dose, 25 mg) (Supplementary Fig. 2a).^18,23^ Our data indicated that dose-escalation OIT efficiently inhibited watery allergic diarrhea in this murine model (Supplementary Fig. 2b and c).

**Fig. 1:**
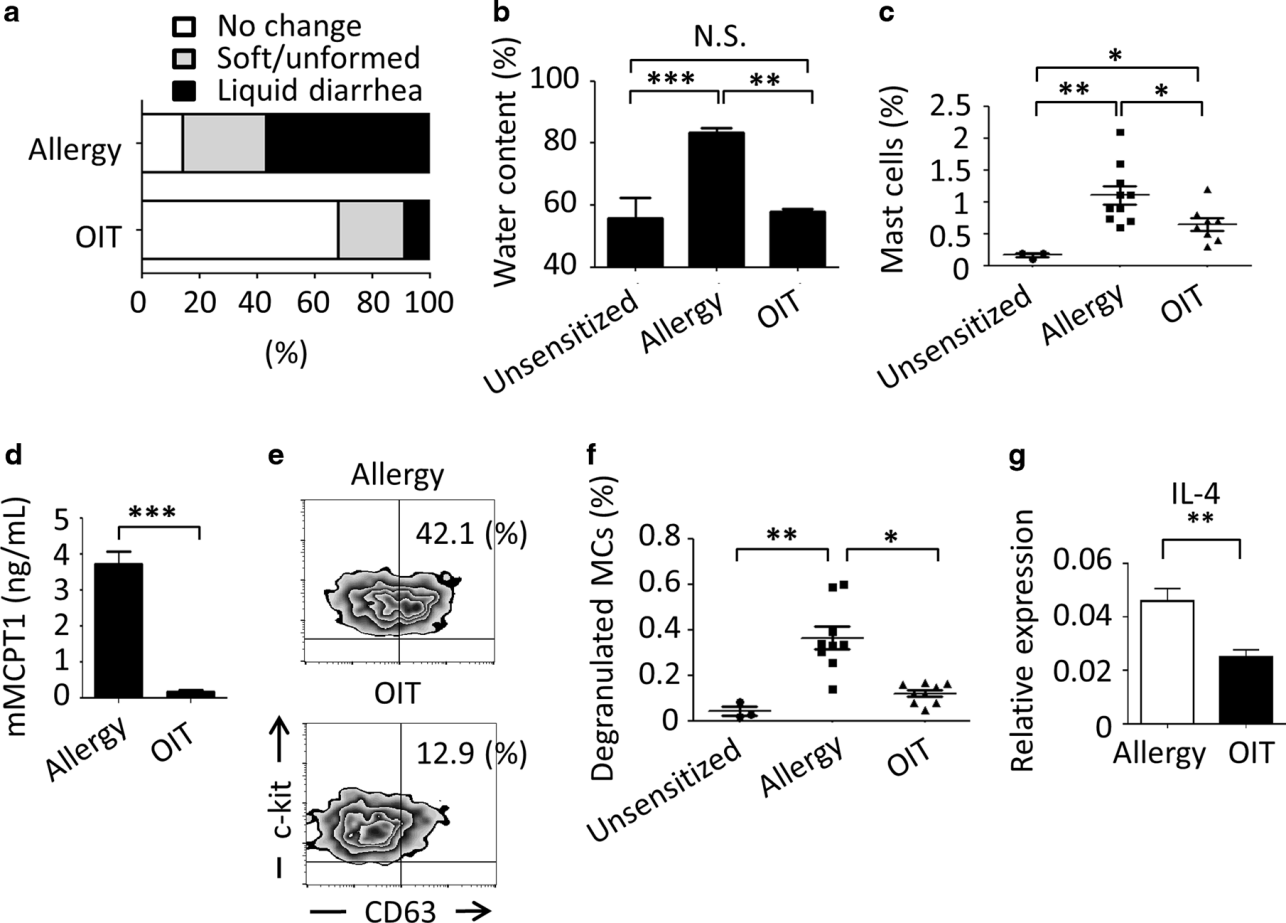
Oral immunotherapy (OIT) desensitizes mast cells in local intestinal mucosa. **a** BALB/c mice showing allergic diarrhea were treated by OIT protocols or left untreated and the status of their stools was evaluated (Allergy, *n* = 15; OIT *n* = 22). **b** The water content of the stools was measured (Unsensitized, *n* = 3; Allergy, *n* = 6; OIT, *n* = 4), **P < 0.01, *** *P* < 0.001. N.S. indicates not significant. **c** Percentages of colonic MCs were examined by flow cytometry. Cells were gated on CD45^+^ c-kit^+^ FcεRIα^+^ cells. Percentages of MCs among CD45^+^ cells are shown (Non-allergic, *n* = 3; Allergy, *n* = 10; OIT, *n* = 8). *P < 0.05, ** *P* < 0.01. **d** Serum mouse mast cell protease-1 (mMCPT1) concentrations were measured by ELISA. (Allergy, *n* = 5; OIT, *n* = 4). **e** CD63 expression on colonic MCs was examined by flow cytometry. Cells were gated on c-kit^+^ and FcεRIα^+^ cells and the percentages of CD63^+^ MCs among all MCs were determined by flow cytometry. Data are representative of nine mice. **f** CD63^+^ MCs among CD45^+^ cells are shown (Intact, *n* = 3; Allergy, *n* = 9; OIT, *n* = 9). *P < 0.05, ** *P* < 0.01. **g** IL-4 cytokine expression was analyzed by quantitative RT-PCR. Each result was normalized against the expression of *Gapdh*. Data are shown as means ± SEM, *P < 0.05. N.S., not significant.

We further categorized the severity of antigen-induced allergic diarrhea according to three clinical criteria—no change, soft/unformed, and liquid diarrhea—on the basis of the water content (Supplementary Fig. 2d and e) and evaluated in detail the efficacy of successful OIT treatment (Fig. 1). In the dose-escalation, OIT group watery diarrhea development was inhibited upon allergen oral challenge, and about 70% of mice showed no change in the feces (Fig. 1a and b). In contrast, over 80% of mice in which allergy was provoked and OIT was not given developed soft to liquid diarrhea. Furthermore, the percentage of MCs was significantly lower in the OIT group than in the allergy group (*P* < 0.05, Fig. 1c). But notably there were still significantly more MCs in these desensitized mice than in the intact group, our data indicated that the dose-escalation OIT protocol effectively created a desensitized state and thus prevented the development of orally induced allergic signs (i.e., severe diarrhea).

### Induction of orally desensitized mucosal MCs

We next analyzed the activation status of intestinal MCs upon OIT. Levels of serum mast cell protease 1, which are correlated with the activation status of mucosal MCs,^24^ were significantly lower in OIT mice than in untreated mice (*P* < 0.01, Fig. 1d). When gastrointestinal MCs were isolated and examined, the expression level of CD63, associated with granule release,^25^ was also lower in the OIT group than in mice with allergic diarrhea (Fig. 1e). Furthermore, the frequency of degranulated MCs was significantly decreased to the normal level after OIT (Fig. 1f). Because IL-4 produced by MCs plays a pathological role in enhancing Th2-type responses,^10^ we sorted MCs from allergic and OIT mice and compared their gene expression of IL-4 (Fig. 1g). MCs from mice given OIT had significantly lower expression of *Il4* than those from allergic mice (Fig. 1g). Thus, desensitized and reduced numbers of IL-4-producing MCs were found in the gastrointestinal tract of OIT group compared with the allergic group, suggesting that OIT directly regulates local MCs and thus inhibits their acquisition of pathological characteristics. These results further suggest that orally administered allergen can directly control and desensitize pathogenic MCs.

### Simultaneous induction of regulatory T-cells and desensitized MCs by OIT

**Fig. 2:**
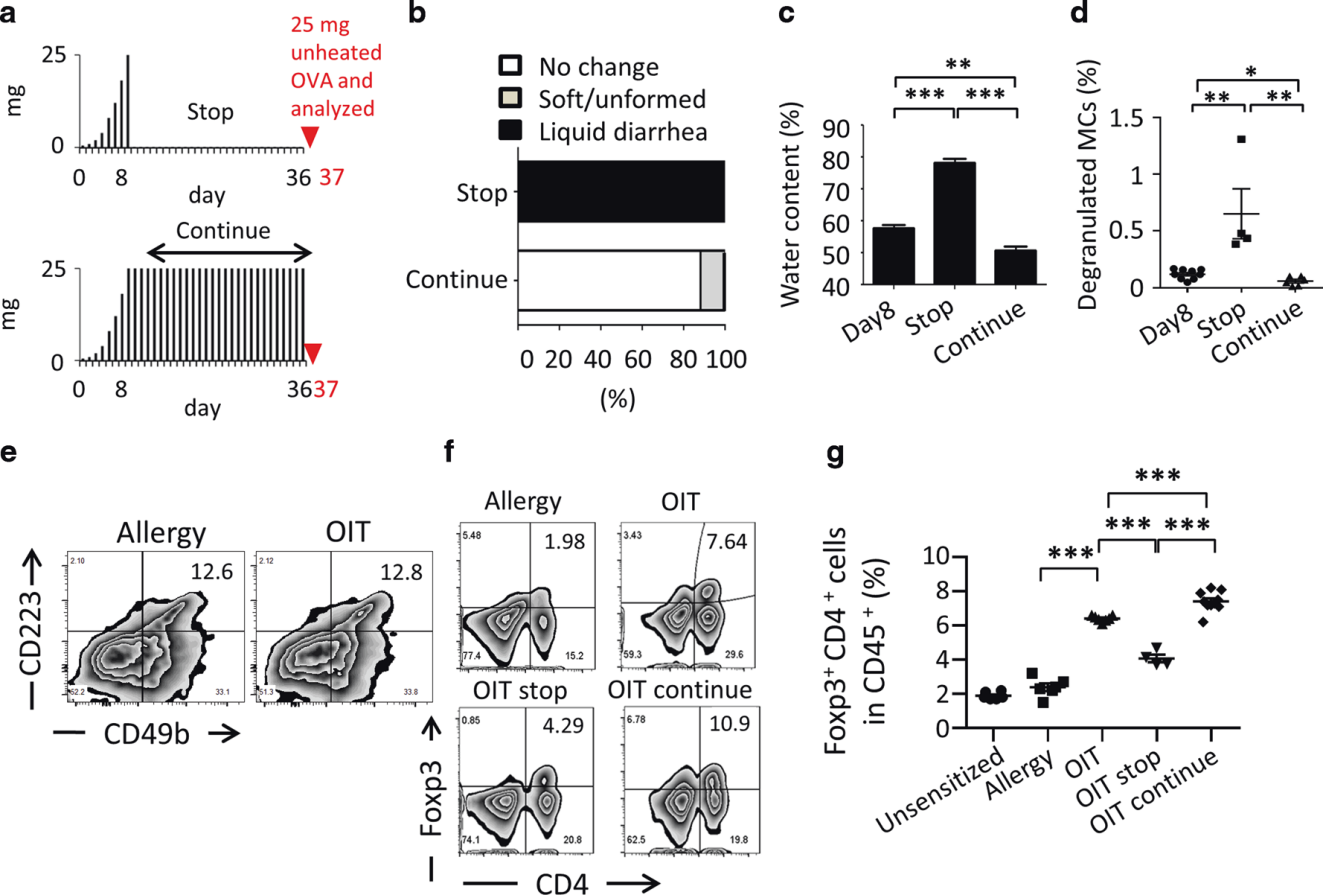
Continuous exposure to allergen after successful oral immunotherapy (OIT) sustains the reduction in diarrhea and the regulatory T-cell response. **a** The protocol used in this experiment. Black bars indicate the amounts (mg) of heated ovalbumin (OVA), and the red triangle indicates the oral administration of 25 mg unheated OVA. **b** Status of mice stools upon unheated OVA administration was evaluated. (Stop OIT, *n* = 4; continue OIT, *n* = 8). **c** Water content of stools was measured (OIT [day 8], *n* = 4; stop [day 37], *n* = 4; continue: *n* = 4 [day 37]). Data are shown as means ± SEM, **P < 0.01, ***P < 0.001. **d** CD63^+^ intestinal mast cells as a percentage of all CD45^+^ cells are shown (day 8 of OIT, *n* = 9; day 37 of stop, *n* = 4; continue, *n* = 4). **e** Cells were harvested from the colon and stained to evaluate the presence of Tr1 cells. Cells were gated on CD4. Number indicates percentage of CD223^+^ CD49b^+^ Tr1 cells. Data are representative of at least three individuals. **f** and **g** Cells were gated on CD45 and percentages of Foxp3^+^ CD4^+^ T cells are shown. Representative FACS plots were shown and each dot represents an individual mouse. Data are shown as means ± SEM, ***P < 0.001.

Upon dose-escalation OIT, continuous administration of allergen is generally required for maintenance of oral unresponsiveness and continuing inhibition of allergic signs.^13^ We therefore expected that continuous administration of allergen would also be required for maintenance of desensitization of MCs, so we examined whether continued or discontinued oral administration of allergen was required for the maintenance phase of OIT (Fig. 2a). When OVA administration to OIT groups was stopped after desensitization had been induced by the initial OIT treatment, mice showed a reoccurrence of allergic diarrhea, which was correlated with a significant increase in the percentage of CD63^+^ degranulated MCs (Fig. 2b–d). In contrast, continuous OVA administration consistently suppressed allergic watery diarrhea and maintained the desensitized status of MC degranulation (Fig. 2d). These results clearly indicated that continuous oral administration of allergen was indispensable for the maintenance of desensitized MCs in the intestinal tract.

OIT is known to induce the production of T cells with suppressor functions, such as Treg and Tr1 cells.^26,27^ In addition to the induction of desensitized MCs by OIT, our results suggested that dose-escalation OIT would result in the generation of these regulatory-type T cells. We therefore next examined the numbers of these two T-cell subsets. Although Tr1 cells with phenotypic characteristics of CD49b^+^ CD223^+^ and CD4^+^ are involved in oral unresponsiveness,^26^ we found no alteration in the numbers of Tr1 cells in the OIT group compared with the untreated allergy group (Fig. 2e). In contrast, the percentage of Foxp3^+^ Treg cells was significantly increased in the OIT group compared with the allergy groups and then significantly reduced compared with the OIT group when oral administration of allergen was stopped (Fig. 2f and g). In our previous study, adoptive transfer of intestinal CD25^+^ CD4^+^ Treg cells to food-allergic mice suppressed allergic diarrhea, thus emphasizing the critical role of Treg cells in controlling the allergic symptom.^28^

**Fig. 3:**
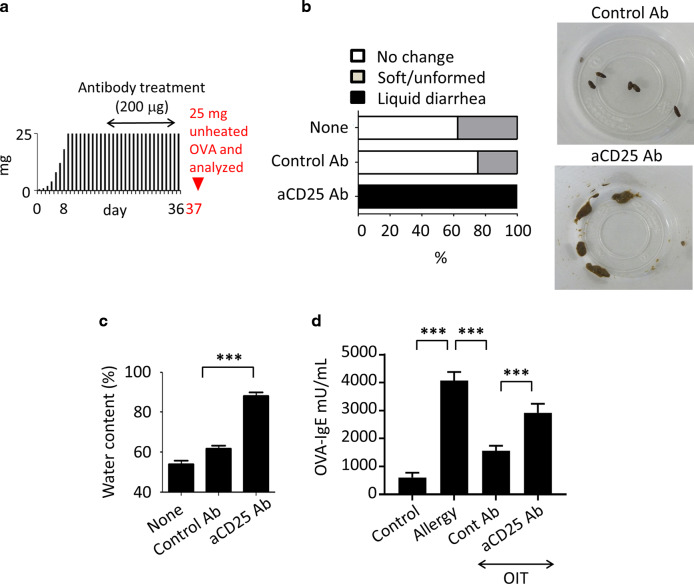
Regulatory T cells are required for treatment of food allergy. **a** Experimental scheme. Black bars indicate the amounts (mg) of heated ovalbumin (OVA), and the red arrowhead indicates initiation of daily oral administration of 25 mg unheated OVA. **b** Mice receiving oral immunotherapy were treated with 100 µg of anti-CD25 monoclonal antibody (aCD25) or left untreated (None) or treated with control antibody. Stool status was then evaluated, *n* = 3. Data were representative of two independent experiments. **c** The water content of the stools in each treatment group was measured. Data are shown as means ± SEM of *n* = 3, ***P < 0.001. **d** Serum OVA-specific IgE was measured by using ELISA. Data are shown as means ± SEM of *n* = 4–6, ***P < 0.001.

To further confirm the requirement for Treg cells in the effectiveness of OIT, we gave anti-CD25 monoclonal antibody^29^ or control rat IgG Ab to OIT-treated mice (Fig. 3a). All mice that received anti-CD25 mAb, resulted in partial depletion of Treg from mucosal compartment (data not shown), showed a recurrence of allergic diarrhea (Fig. 3b and c). In addition, anti-CD25 mAb treatment enhanced OVA-specific IgE production in OIT-treated mice; prior to anti-CD25 treatment, IgE antibody levels had been low (Fig. 3d).

Concomitantly to the expansion of mucosal Treg cells, we found expansion of the systemic Treg population in the peripheral blood and spleens of mice that received OIT treatment (Supplementary Fig. 3). These results indicate that OIT-mediated expansion of the circulating Treg population (possibly derived from mucosal compartments) contributes to the suppression of allergic reactions (e.g., systemic and skin symptoms); this suppression occurs through the reduction of both allergen-specific IgE production and degranulation of MCs and basophils (Fig. 3d and Supplementary Fig. 3).

### Desensitized MCs play a critical role in the regulation of allergic signs

**Fig. 4:**
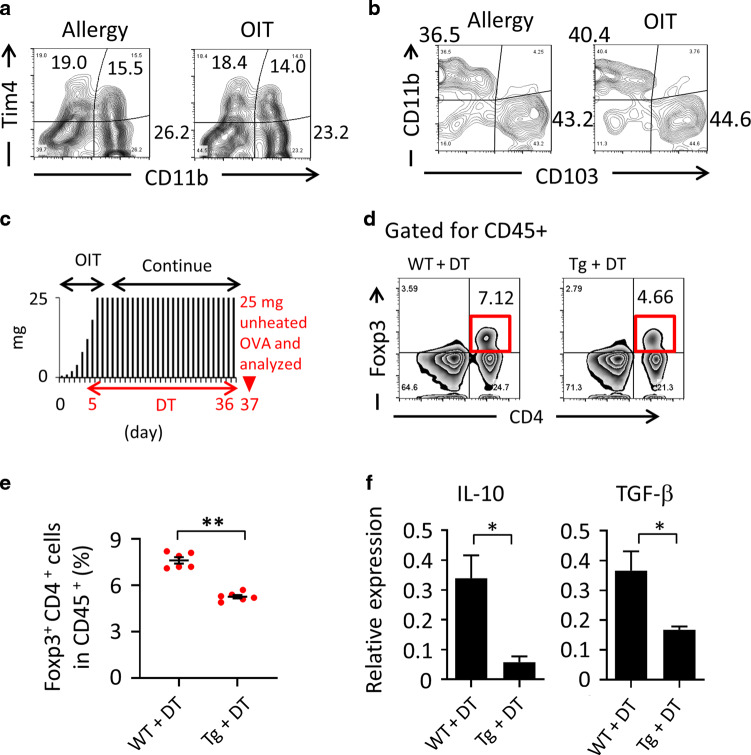
Involvement of mast cells in expansion of Foxp3 Treg populations upon oral immunotherapy (OIT). **a** Tim4 and CD11b and (**b**) CD11b and CD103 were both gated for CD11c^+^ CD45^+^ and examined by flow cytometry. The numbers shown indicate the percentages of cell populations. Data represent three individual experiments. **c** Protocol used in this experiment. Black bars indicate the doses of heated ovalbumin (OVA), and the red triangle indicates the administration of 25 mg of unheated OVA. Diphtheria toxin (DT) was administered to Mas-TRECK mice during OIT. **d** and **e** OIT mice (wild-type [WT] or Mas-TRECK transgenic [tg] mice) received DT from days 5 through 36, and Foxp3-expressing Tregs were observed by flow cytometry on day 37 upon inoculation of unheated 25 mg of OVA. Data are gated for CD45 and representative of at least three individual experiments. Data are shown as means ± SEM, **P < 0.01. **f** CD25^+^ CD39^+^ CD103^+^ CD4^+^ T cells were isolated, and IL-10 and TGF-β gene expression was analyzed by quantitative RT-PCR. Data are shown as means ± SEM, *P < 0.05 (all groups, *n* = 4).

In addition to the generation of desensitized MCs (Fig. 1), we found that OIT induced the production of Treg cells for the suppression of allergic diarrhea (Fig. 2f, g and 3). However, the mechanisms behind the relationship between the induction of desensitized MCs and Treg cells in OIT needed to be further elucidated. An increase in the induction of tolerogenic dendritic cells (DCs) plays important role in the establishment of oral unresponsiveness.^30^ We therefore considered it important to elucidate whether DCs were involved in the increased production of Treg cells via desensitization of MCs. We first examined the possible involvement of desensitized MCs in the induction of DCs or increase in their populations, because MCs have been shown to play a role in the induction of tolerogenic DCs in tissue transplantation mouse model.^31,32^ We therefore examined the profiling of these DC subsets, such as Tim4^+^ CD11c^+^ DCs, including pro-allergic subsets, and CD103^+^ CD11b^neg^ CD11c^+^ tolerogenic DCs.^33,34^ Both OIT and allergic mice and found that the ratios of DC subsets did not differ significantly between the two groups of mice (Fig. 4a and b). Because our findings suggested that the properties of intestinal DCs were not altered by OIT, alternative pathways—including MC-mediated direct pathways—might be involved.

The novel properties of MCs in promoting Treg-cell-mediated suppression of papain-induced allergic inflammation have recently been elucidated.^20^ Accumulated evidence has further revealed the tolerogenic properties of MCs in any diseases (e.g., atopic dermatitis, asthma and graft-versus-host disease).^35,36^ Therefore, next, we examined whether desensitized MCs directly mediated Treg induction. We depleted MCs in Mas-TRECK mice during the OIT procedure (days 5 through 36) by giving diphtheria toxin (see Fig. 4c). Under normal conditions or in the allergic state, MC depletion did not reduce the percentage of Treg cells in the intestinal tract (Supplementary Fig. 4a); however, depletion of MCs during OIT led to a significant reduction in the percentage of Treg cells in the intestine compared with control group (*P* < 0.01, Fig. 4d and e), but not in the spleen and mesenteric lymph nodes (Supplementary Fig. 4b). Importantly, comparison of the quality of the Treg cells of OIT mice with and without MCs showed significant reductions in the production of inhibitory cytokines in the latter (e.g., IL-10 and TGF-β) (Fig. 4f). These findings suggested that OIT induces desensitized MCs, which mediate the induction and maintenance of Treg cells possessing suppressive properties such as inhibitory cytokine synthesis.

### Functional modulation of pathogenic MCs by the desensitization process

**Fig. 5:**
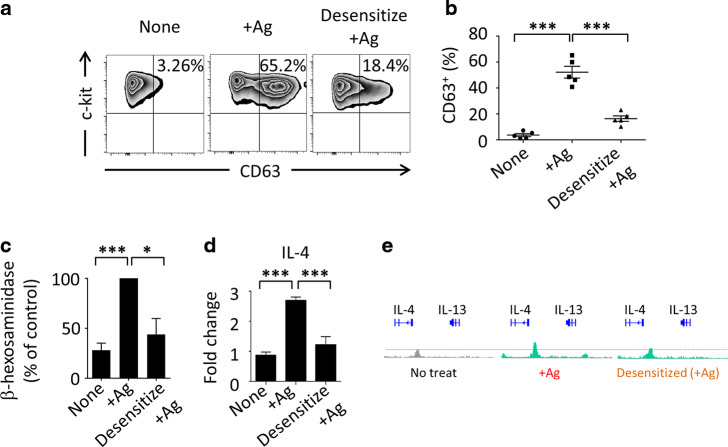
Rapid desensitization controls pathogenic mast cell (MC)-mediated Th2 immunity. **a** and **b** DNP-IgE bound bone marrow MCs (BMMCs) were stimulated or desensitized with antigen (DNP-HSA). CD63 expression was observed by fluorescence-activated cell sorting. Percentages indicate CD63^+^ in whole BMMCs. *** *P* < 0.001. **c** A β-hexosaminidase assay was performed. Data are expressed as differences from the value in undesensitized activated MCs (100%) as a control. *P < 0.01, *** *P* < 0.001. **d** IL-4 expression was examined by quantitative RT-PCR. Data are expressed as differences from non-activated or desensitized MCs (“None”) as a control (**e**) Chromatin immunoprecipitation was performed; acetylation levels of H3K27 of IL-4 and IL-13 promoters are shown.

To further elucidate whether OIT-induced desensitized MCs are involved in the induction and maintenance of Treg cells, we adopted an in vitro desensitization protocol.^37,38^ To generate desensitized MCs, about 95% purified BMMC were utilized (Supplementary Fig. 5a and b) and IgE-bound BMMCs were prepared and treated with gradually increasing amounts of allergen, or model antigen (2,4-dinitrophenyl; DNP). The treatment induced desensitized MCs, because reduced surface expression of the degranulation-associated molecule CD63 and reduced release of degranulation-associated β-hexosaminidase compared with those in untreated allergic mice were noted (Fig. 5a–c).^25^ These results are consistent with our in vivo data that OIT-desensitized MCs efficiently reduced surface CD63 expression (Fig. 1e and f).

As described above, not only degranulation of MCs but also the control of pathogenic Th2 responses mediated by elevated IL-4 production by these MCs (Fig. 1g) was important for allergy treatment. To further confirm this point, we examined IL-4 expression levels in MCs desensitized by OIT and compared them with those in degranulated MCs from mice with allergic diarrhea. IL-4 expression by the desensitized MCs was significantly reduced by the OIT process (Fig. 5d). We further evaluated the shift in MCs from pathogenic (or pro-allergic) status to regulatory (desensitization) status by evaluating the histone modification status at Th2 cytokine gene loci—especially those encoding IL-4 and IL-13 (Fig. 5e). MCs were isolated from normal mice, mice with allergic diarrhea, and OIT mice. Control, activated, and desensitized MCs were examined (Fig. 5e). ChIP-Seq analysis revealed that the levels of histone H3 acetylated at Lys27 (H3K27Ac) were decreased around the promoter regions of the *Il4* and *Il13* gene loci after desensitization (Fig. 5e). This evidence clearly indicated that the in vitro desensitization process functionally and directly regulated degranulation of, as well as Th2 cytokine production by, MCs. These results imply that the desensitization procedure modulates allergic Th2-prone MCs to acquire unique, predictively immunoregulatory characteristics that promote clinical unresponsiveness.

### Pivotal roles of desensitized MCs in expansion of Treg cell populations in an IL-2 dependent manner

On the basis of our in vitro and in vivo observations of a functional shift in MCs, which we predicted would be the acquisition of a regulatory function upon OIT (Figs. 4 and 5), we adopted an in vitro desensitization protocol^37,38^ to directly reveal Treg-cell expansion by OIT-induced desensitized MCs. To generate desensitized MCs, IgE-bound bone marrow (BM) MCs were prepared and treated with gradually increasing amounts of allergen.

**Fig. 6:**
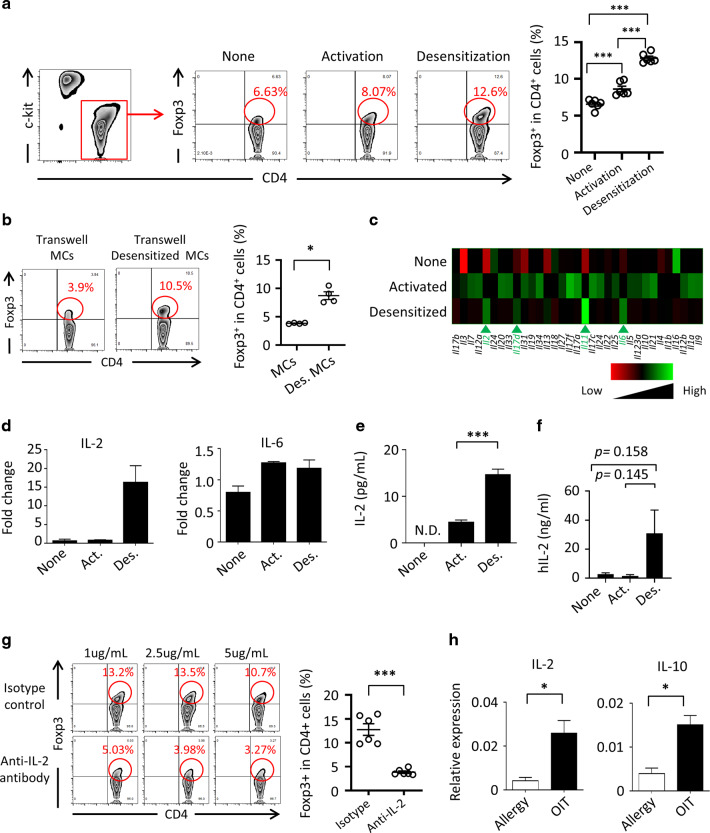
Foxp3 Treg expansion by desensitized regulatory mast cells. **a** DNP-IgE bound (None), antigen-stimulated (Activation), or desensitized (Desensitization) bone marrow MCs (BMMCs) were co-cultured with CD4^+^ T cells and expansion of the Foxp3^+^ CD4^+^ T cell population was analyzed by flow cytometry and cell sorting. Numbers indicate percentages of Foxp3^+^ among all CD4^+^ T cells. **b** IgE-bound and desensitized BMMCs and CD4^+^ T cells were co-cultured under separated conditions in transwells. Percentages of Foxp3^+^ cells among all CD4^+^ T cells are shown. **c** Gene microarray was performed with DNP-IgE bound (None), antigen-stimulated (Activated), and desensitized BMMCs. Shown is a heatmap representative of entities termed cytokines. **d** Expression of the genes encoding IL-2 and IL-6 was analyzed by quantitative RT-PCR. Data are shown as means ± SEM. Act., activated; Des., desensitized. **e** IL-2 production was analyzed by ELISA. Data are shown as means ± SEM. ***P < 0.001 (all groups, *n* = 5). **f** IL-2 production from hPBMC was analyzed by ELISA. Data are shown as means ± SEM; all groups, *n* = 3. **g** Percentages of Foxp3^+^ cells in coculture of desensitized BMMCs are shown. Isotype or anti-IL-2 antibody was added. **h** Gene expression of in vivo sorted MCs was analyzed by quantitative RT-PCR. Each result was normalized against the expression of *Gapdh*. Data are shown as means ± SEM, ***P < 0.01, *P < 0.05.

Because our results had suggested that OIT-induced desensitized MCs were involved in the expansion of Treg cells (Fig. 4), this experiment aimed to directly address whether the desensitized MCs could expand Treg cells in an in vitro coculture system. CD4^+^ T cells were cultured with antigen -desensitized or allergic BMMCs (Fig. 6a). Foxp3 expression by these CD4^+^ T cells was then examined by FACS (Fig. 6a). Desensitized MCs, but not control or allergic MCs, expanded the Treg cell population (Fig. 6a). As reported previously, MCs lack the ability to present antigens.^20^ Indeed, desensitized MCs lacked the expression of MHC-II and co-stimulatory molecules (e.g., CD80), and their ability to induce the differentiation of Treg cells from naïve CD4^+^ T cells was limited (Supplementary Fig. 6 and 7). These results suggest that secretory factors are involved in expansion of the Treg cell population by desensitized MCs. To explore this possibility, we performed a coculture experiment with a transwell assay (Fig. 6b). The assay revealed that secreting molecules from the desensitized regulatory MCs were involved in the induction of Treg cells, because physical separation of the desensitized MCs and the CD4^+^ T cells still resulted in the expansion of Treg cells (Fig. 6b).

To examine the molecular mechanisms underlying Treg induction by the desensitized MCs, we performed gene profiling of in vitro*-*activated and -desensitized MCs (Fig. 6c). Expression of the genes encoding several cytokines (IL-2, IL-6, IL-11 and IL-17d) was increased in the desensitized MCs (Fig. 6c). Furthermore, when we examined the actual production of these cytokines, the production of IL-2, but no other cytokines, was significantly increased by the desensitized regulatory MCs (Fig. 6d, e and data not shown). Importantly, we harvested MCs from human peripheral blood, desensitized them in vitro, and then assessed their production of IL-2 (Fig. 6f). These results revealed a trend (*P* = 0.145) of increased IL-2 production from desensitized human MCs when compared with sensitized human MCs. IL-2’s roles in the induction and proliferation of Treg cells are well-known;^39,40^ therefore, next, we conducted IL-2 neutralizing experiments using cocultures of desensitized murine regulatory MCs and CD4^+^ T cells.^41^ Anti-IL-2 neutralizing mAb greatly inhibited Treg-cell induction by desensitized regulatory MCs in vitro (Fig. 6g). These results clearly indicated that the desensitized regulatory MC–IL-2 pathway plays a critical role in Treg-cell induction in OIT-induced suppression (Fig. 4). Finally, we examined the expression of these regulatory cytokines by MCs isolated from the intestines of allergic and OIT mice. Importantly, quantitative RT-PCR analysis revealed significantly increased expression of IL-2 and IL-10 in orally desensitized lamina propria MCs (*P* < 0.05, Fig. 6h).

## Discussion

OIT is an efficient desensitization treatment for food allergy.^14,15^ In the early stage of OIT, gradual dose escalation is required to suppress the degranulation of MCs and basophils.^17^ Previous studies have executed the OIT protocol before the onset of food allergy (i.e., in the pre-sensitization phase or naïve stage), thus leading to the induction of oral tolerance.^42,43^ However, in clinical practice, most patients are already sensitized and susceptible to food allergy,^13–15^ and OIT has only infrequently been used in food-allergic patients.^13–15^ Therefore, to understand the mechanistic relevance of allergy treatment through OIT in humans, it is important to develop an OIT treatment model for food allergy that better resembles the clinical situation.

Here, by developing a clinically relevant OIT murine model, we revealed a novel mechanistic immunological process in the intestinal mucosa upon successful OIT. Our OIT model indicated that dose escalation was more efficient than defined-dose treatment in desensitizing MCs (Supplementary Fig. 2). The functional characteristics of desensitized and immunosuppressive MCs had not been well studied, so we found and scrutinized the unique characteristics of desensitized MCs with regulatory functions (regulatory MCs) that play a major role in the establishment of oral unresponsiveness by OIT.

First, we revealed the importance of mucosal Treg cells in OIT. The relationship between increased Treg cell populations and successful OIT has been well documented; however, direct evidence of the requirement for Treg cells in preventing food allergy had not been presented. Here, by antibody depletion of both antigen-specific and -nonspecific populations, we examined the indispensable roles of Treg cells and clearly found that all mice developed severe diarrhea upon the reduction of Treg cell numbers (Fig. 3). In the context of Treg cell expansion, an increase in Treg cell numbers by the administration of low-dose IL-2 efficiently suppresses both allergy and auto-immune disease.^44,45^ Daily intraperitoneal injection of IL-2 for 5 days successfully expands the Treg cell population without affecting effector T cells.^44^ Our study revealed an increase in local IL-2-producing desensitized (or regulatory) MCs upon OIT, whereas a decrease in the abundance of these MCs simultaneously decreased Treg cell abundance at the local mucosa. These findings imply that continuous and optimal-dose antigen delivery by OIT leads to the induction of IL-2-producing regulatory MCs for efficient expansion of Treg cell populations. Despite the considerable debate regarding the functional and transcriptional heterogeneity and functional instability of Treg cells, these properties have important implications for Treg cell-based immunotherapy,^46^ particularly given that effective induction and maintenance of Treg cells is important for the acquisition of unresponsiveness in various allergic diseases, such as allergic rhinitis.^13^

In our current study, anti-CD25 antibody treatment in OIT mice led to the elimination of Tregs and thus the recurrence of allergic reactions and increased OVA-IgE production (Fig. 3). It is plausible that IL-10 and TGFβ derived from Treg cells directly suppress function of IgE-producing cells or remaining undesensitized MC populations still residing in the OIT-treated mucosa, so that anti-CD25 antibody treatment leads to the gastrointestinal sign of watery allergic diarrhea (Fig. 3e).

To further understand the role of the Treg network, we assessed possible crosstalk between Treg cells and T follicular-cell populations including TFH, TFR, and TFH13, which have been shown to important in the control of IgE production.^47,48^ We found no significant changes in the T follicular-cell population in OIT mice treated with anti-CD25 antibody compared with control antibody (Supplementary Fig. 8a and b), indicating that Tregs had little or no effect on the expansion of T follicular-cells in this model. Likewise, MC-depleted mice lacked significant changes in the T follicular-cell population (Supplementary Fig. 8a and b), demonstrating that the desensitized MC–Treg pathway dose not contribute to T follicular-cell -mediated IgE production in this model.

Our current results indicate that suppression of Th2 cytokines through desensitization occurs simultaneously with the upregulation of IL-2 and IL-10 by desensitized (or regulatory) MCs. In some circumstances (e.g., dermatitis, asthma, and graft-versus-host disease), MCs possess immunosuppressive properties mediated by the production of immunoregulatory cytokines such as IL-2, IL-10, IL-33, and TGF-β.^19,20^ Considering these previous results together with our current ones (Fig. 6), it is plausible to suggest that desensitized MCs behave as a form of regulatory cells producing these regulatory cytokines and categorized as regulatory MCs, and that they directly or indirectly (via the induction of Treg cells) suppress undesired allergic responses. MCs have also been shown to have a direct suppressive function, which is mediated in an IL-10-dependent manner.^19^ Skin allograft rejection is regulated by IL-10 derived from skin MCs.^19^ IL-10 derived from skin MCs educates DCs to induce Tregs.^19^ IL-10 from MCs is also involved in the maintenance of immune privilege in the bladder.^49^ These findings suggest that IL-10-producing MCs can behave as regulatory MCs for the control of undesired immune responses. We found here that, along with IL-2 expression, IL-10 expression was upregulated in OIT-treated MCs (Fig. 6g); thus, IL-2 and IL-10 dual synthesis in response to OIT-induced mucosal desensitization of MCs resulted in a duplex suppressive pathway of induction of Treg cells and direct inhibition of Th2 pathway for the efficient induction of oral unresponsiveness against food allergy. However, in in vitro desensitized MCs we were unable to find upregulation of IL-10 (data not shown). In relation to this, it has been shown that IgG1–FcγRIII pathways induce IL-10 production by MCs in contact dermatitis.^50^ IL-4 stimulation enhances the production levels of FcγRIII and crosslinking with antigen-specific IgG1 induces IL-10 production.^50,51^ Both allergen-specific IgE and IgG antibodies have been at the center of discussions of the desensitization of MCs.^52^ An increase in the levels of allergen-specific IgG antibodies has been suggested to be associated with the desensitization of allergic responses.^42^ Indeed, the administration of allergen-specific IgG antibodies to a mouse model of food allergy suppressed MC activation via FcγRIIb.^42^ The involvement of IgG–FcγRIIb or FcγRIII pathways in OIT in vivo cannot be ignored in terms of both the inhibition of MC degranulation and the production of IL-10 by MCs.

In an airway inflammation model, IL-33 stimulation induced IL-2 production by MCs and resulted in limitation of inflammation.^20^ In a skin inflammation model, IL-2 release by MCs was induced by IL-33 and further enhanced by IgE stimulation.^35^ However, mice in an IL-33-deficiency food allergy model had less severe signs than WT mice, indicating that IL-33 causes allergy rather than suppression in food allergy.^53^ IL-33 promotes food anaphylaxis in epicutaneously sensitized mice by targeting MCs.^53^ Here, we found no difference in IL-33 expression between allergic and OIT-treated intestinal MCs (Supplementary Fig. 9a). Indeed, we further examined this point by utilizing IL-33-deficient MCs and CD4^+^ T cells that were deficient in ST2, the receptor for IL-33 (Supplementary Fig. 6b). Moreover, coculture of desensitized IL-33-deficient MCs with CD4^+^ T cells still induced Foxp3^+^ Tregs. Thus, the induction of Tregs by desensitized regulatory MCs occurred via an IL-2-dependent but IL-33-independent pathway (Supplementary Fig. 9b).

The molecular mechanisms underlying desensitization-induced IL-2 production was not fully elucidated here. It has recently been found that desensitization of MCs inhibits calcium flux and suppress degranulation, with modulation of the cytoskeleton.^54^ In our recent and separate study, we also found partial internalization of surface IgE and its receptor FcεRI by desensitization in vitro (data not shown); however, this reduced amount of FcεRI is still capable of transducing signals.^54^ This implies that continuous stimulation by Ag–IgE complex modulates FcεRI signal transduction, resulting in a functional change in MCs. Taken together, these pieces of evidence imply the existence of unique and as-yet-unelucidated cascades that regulate MC functions, including the shift from pathogenic to regulatory behaviors and the consequent induction of IL-2 and IL-10, producing “regulatory MCs” via constant stimulation from IgE and its receptor pathway. In addition, accumulated evidence from other studies and ours reveals that inflammatory cytokines from MCs are involved in the onset of chronic inflammatory disorders in various tissues and organs.^25,55^ The conversion of MC function from pathogenic to regulatory might be a critical approach to curing chronic inflammation beyond allergic diseases. Further analysis is therefore required to uncover the important role of allergen–IgE-complex-mediated signaling in the conversion from pathogenic to beneficial MCs.

In summary, our results demonstrated that activated and degranulated MCs were essential pathological elements for the development of allergic diarrhea. However, OIT with an antigen-dose-escalation protocol effectively induced oral unresponsiveness against allergen-induced allergic diarrhea by the conversion of activated pathogenic MCs to desensitized regulatory MCs. Thus, OIT caused desensitized regulatory MCs to produce IL-2 for the expansion of Treg cell populations and IL-10 for the inhibition of allergic responses, whereas pathogenic Th2 cytokine production was inhibited (Supplementary Fig. 11). Our findings provide new insights into the pathogenic and beneficial aspects of MCs, which can be manipulated by the appropriate form of OIT to control allergic responses, including allergic diarrhea, through the coordinated cellular cascade of regulatory MCs and Treg cells.

## Materials and methods

### Mice

Female mice (age, 7–10 weeks) were used. BALB/c mice were purchased from CLEA Japan (Tokyo, Japan). Mas-TRECK mice (mice with deletion of MCs via IL4 enhancer elements upon diphtheria toxin treatment) were gifted as previously described.^22^ BALB/c background IL-33- and ST2-deficient mice were a gift from Dr. S. Akira (Osaka University). MC-deficient *Kit*^*W-sh/W-sh*^ mice were obtained from Dr. H. Suto (Atopy Research Center, Juntendo University, Japan) and backcrossed at least seven times on a BALB/c background. All mice were maintained under specific-pathogen-free conditions at the experimental animal facility of the institutes. All experiments were approved by the Animal Care and Use Committee of the University of Tokyo and Chiba University.

### Food allergy and OIT

Mice were pre-sensitized with 1 mg of ovalbumin (OVA) (Fraction V, Sigma Chemical Co., St. Louis, MO, USA) in complete Freund’s adjuvant (CFA, Difco Laboratories, Detroit, MI, USA) by subcutaneous injection as described previously.^56^ After 1 week, the sensitized mice were challenged three times a week for several weeks with 50 mg of orally administered OVA in phosphate-buffered saline (PBS). In this state, the mice were defined as the “allergy group.” Mas-TRECK mice were injected with 150 µL of 1 µg/mL diphtheria toxin (Sigma-Aldrich, St Louis, MO, USA) intraperitoneally for 5 consecutive days and continued every other day as described previously.^25^ All mice used in the OIT procedure were first confirmed to show allergic diarrhea upon oral challenge with 25 mg raw OVA. The next day, OIT was performed by using the following protocols. In dose-escalation OIT, we used a modified previously reported protocol^57,58^ to develop an OIT murine model that more closely resembled the human clinical scenario. OVA was heated to 100 °C for 5 min. Increasing doses of heated OVA in PBS were given intragastrically daily for 8 days, at 0.5 mg (day 1), 1 mg (day 2), 2 mg (day 3), 4 mg (day 4), 8 mg (day 5), 12 mg (day 6), 18 mg (day 7), and 25 mg (day 8); thereafter mice daily received 25 mg of unheated OVA during the maintenance phase (36 days). Mice that had completed OIT were defined as the “OIT group”.

Stool status was evaluated by using an appearance scale and by quantifying the water content (see Supplementary Fig. 2d and e). Water content was determined by measuring the difference between the initial fecal weight and the dry weight.^59^ We putted the feces in closed container with silicagel, and the dry weight was measured on the fifth day.

### Cell collection and fluorescence-activated cell sorting (FACS) analysis

Mononuclear cells were isolated from colonic lamina propria and Peyer’s patches, as previously described.^25^ Briefly, epithelium was dissociated by using 0.5 mM EDTA and the tissues were further treated with 1.25 mg/mL of collagenase at 37 °C.^25^ To collect mononuclear cells from mesenteric lymph nodes and spleen, the tissues were mashed mechanically and filtered through 70 μm mesh. Lymphocyte separation medium (MP Biomedicals, Santa Ana, CA, USA) was used to isolate peripheral blood mononuclear cells (PBMCs).

For flow cytometric analysis, cells were incubated with 5 µg/mL of an anti-CD16/32 antibody (Fc block, BD Pharmingen, San Diego, CA) for 5 min and stained for 30 min at 4 °C with fluorescence-labeled antibodies (Abs) specific for c-kit (2B8), CD25 (3C7), CD45 (30F-11), CD63 (5A9),^11^ RORγt (Q31–378) (BD Pharmingen), and FcεRIα (MAR-1) and Foxp3 (FJK-16s) (eBioscience, San Diego, CA). CD4 (RM4–5), CD11b (M1/70), CD11c (N418), CD39 (Duha59), CD45 (30F11), CD49b (DX5), CD73 (TY/11.8), CXCR5 (L138D7), PD-1 (RMP1–30), GATA3 (16E10A23), and Tim-4 (F31-5G3) were purchased from BioLegend (San Diego, CA). Cells were analyzed by using FACSCalibur, FACSCanto II, and FACSAria III flow cytometry systems (Becton Dickinson, San Jose, CA, USA). The full gating strategies for MCs is shown in Supplementary Fig. 10.

### Quantitative real-time PCR and microarray analysis

Total RNA was prepared by using TRIzol (Thermo Fisher scientific, Waltham, MA, USA) and reverse-transcribed by using a Superscript VILO cDNA synthesis kit (Invitrogen, Carlsbad, CA, USA), as previously described.^55^ Quantitative reverse transcription–polymerase chain reaction (RT-PCR) was performed with LightCycler 480 II (Roche, Basel, Switzerland) and the Universal Probe Library (Roche). Microarray analysis was performed as described in our previous report.^25^ Briefly, MCs were sorted, and total RNA was extracted from them with TRIzol. cRNA was hybridized with DNA probes on a GeneChip Mouse Genome array (Agilent, Santa Clara, CA, USA). Data were analyzed with GeneSpring software (Agilent).^25^

### In vivo antibody treatment study

Anti-mouse CD25 mAb (PC61, rat IgG1) for cell depletion and rat IgG for control treatment were purchased from Bio X Cell (Boston, MA, USA). Mice were intraperitoneally injected four times (days 9, 13, 17, and 20) with 250 μg of anti-CD25 mAb.

### Cell culture and stimulation

About 95% purity of bone marrow MCs (BMMCs) were obtained as previously described^11^ (Supplementary Fig. 5). Cells were sensitized overnight with anti-dinitrophenyl (DNP) IgE (0.25 μg/mL). The next day, the cells were washed to eliminate the excess of unbound IgE and resuspended at 37 °C in 100 μL of fresh medium with 5 ng/mL IL-3. For desensitization, cells were treated as described in the previous report.^38^ Briefly, DNP-human serum albumin (HSA) was added every 10 min for desensitization in 200 μL culture condition (50 pg, 250 pg, 250 pg, 500 pg, 500 pg, 1 ng, 2 ng, 8 ng, 16 ng, 17.5 ng); the cells were then stimulated with 80 ng for 1 h.^38^ For in vitro coculture analysis, MCs were pre-sensitized with anti-DNP IgE (0.25 μg/mL) overnight without antigen; they were then co-cultured for 3 days at 37 °C in PBS with 8 × 10^5^ CD4 ^+^ T cells isolated from the spleen and mesenteric lymph nodes with MojoSort (Biolegend); the plates were pre-coated with 0.125 µg/mL anti-CD3ε antibody (BD Pharmingen).

### OVA-specific antibody ELISA and cytokine ELISA

For detection of OVA-specific IgE levels in sera, each serum sample was processed with an antigen-specific IgE ELISA kit (Fujifilm, Gunma, Japan) in accordance with the manufacturer’s instructions. Mouse IL-2 production and mouse mast cell protease-1 (mMCP-1) in sera were detected by using the respective ELISA kits (eBioscience, San Diego, CA) in accordance with the manufacturer’s instructions.

### ChIP assay

The antibodies used for chromatin immunoprecipitation (ChIP) assay were anti-trimethylhistone H3-K4 (AR-0169; Bio Rad, Hercules, CA) and anti-acetyl-histone H3-K27 (C15410174; Diagenode, Denville, NJ).

ChIP experiments using anti-trimethylhistone H3-K4, anti-acetylhistone H3-K27, or control Ab were performed with Dynabeads (Invitrogen) as previously described.^60^ In brief, 5 × 10^6^ cells were fixed in 1% paraformaldehyde at 37 °C for 10 min. Cells were sedimented, washed, and lysed with sodium dodecyl sulfate (SDS) lysis buffer (50 mM Tris-HCl, 1% SDS, 10 mM EDTA, 1 mM phenylmethylsulfonyl fluoride (PMSF), 1 mg/mL aprotinin, and 1 mg/mL leupeptin). The lysates were sonicated to reduce the DNA lengths to between 200 and 1000 bp. The soluble fraction was diluted in ChIP dilution buffer and incubated overnight at 4 °C with Ab conjugated with Dynabeads proteins A and G. The immune complexes were then captured by using a magnet and washed with low-salt, high-salt, LiCl, and Tris–EDTA wash buffer. Enriched chromatin fragments were eluted with elution buffer (0.1 M NaHCO_3_ containing 1% SDS). The eluted materials were incubated at 65 °C for 6 h to reverse the formaldehyde cross-links and then treated with RNase A (10 mg/mL) and Proteinase K (40 mg/mL). DNA was extracted with a QIAquick PCR purification kit (Qiagen, Hilden, Germany). The total input DNA (cellular DNA without immunoprecipitation) was purified in parallel. A real-time quantitative PCR analysis was performed by using the StepOnePlus Real-Time PCR System (Thermo Fisher Scientific) via the comparative cycle threshold method with TaqMan probes (Thermo Fisher Scientific) and primers. To calculate the enrichment of each protein to a particular target DNA, we divided the values obtained for each target by the amount of the corresponding target in the input fraction.^60^ All of the results are expressed as percentages of input DNA.

### ChIP-sequencing and Illumina sequencing

Ab-specific immunoprecipitates and total input DNA samples were prepared by using a NEBNext ChIP-Seq [ChIP with massive parallel sequencing] Library Prep Reagent Set for Illumina.^60^ Adaptor-ligated DNA was recovered by using AMPure XP Beads (Beckmancoulter, Brea, CA). This DNA was then amplified by 15 cycles of PCR and again recovered by using AMPure XP Beads. Fifty cycles of sequencing reaction were performed on an Illumina HiSeq 1500 system (Illumina, San Diego, CA). Read sequences (50 bp) were then aligned to the mm10 mouse reference genome (University of California, Santa Cruz, July 2011) by using Bowtie.^61^ Each aligned read sequence was extended to 120 bp to efficiently detect duplicate reads aligned to identical locations. These 120 bp tags were used for further analyses (of BED files). MACS^62^ (model-based analysis of ChIP-Seq) was used for peak calling and visualization of binding, with the parameters set as follows: window size = 300, gap size = 300, and false discovery rate = 0.01.^60^

### Human PBMC analysis

Human peripheral blood CD34^+^ cells (Stem Cell Technologies, Vancouver, British Columbia, Canada) were cultured in serum-free Iscove’s methylcellulose medium (Stem Cell Technologies) and Iscove’s Modified Dulbecco’s Medium containing recombinant (r) human (h) stem cell factor (PeproTech EC, London, England) at 200 ng/mL and rhIL-6 (PeproTech EC) at 50 ng/mL, as previously described.^63^ The number and purity of cultured MCs were confirmed by using FACS.^64^ Cells were stimulated with 0.25 µg/mL of anti-hapten 4-hydroxy-3-nitrophenyl acetyl (Absolute Antibody, Oxford, UK), and increasing doses of NP-BSA (Biosearch Technologies, Novato, CA) (2.5, 12.5, 12.5, 25, 25, 50, 100, 200, 400, 800, and 875 pg) were added every 10 min for desensitization; the cells were then stimulated with 10 ng NP-BSA (Biosearch Technologies) for 1 h and then cultured overnight. IL-2 production in the culture supernatant was detected by using a high-sensitivity human IL-2 ELISA kit (Abcam, Cambridge, UK). These experiments were approved by the Ethics Committee of Juntendo University.

### Statistical analysis

Statistical analysis was performed using the unpaired, two-tailed Student’s *t* test. All statistical analyses were conducted with Prism 7 (GraphPad Software, San Diego, CA, USA). A difference of *P* < 0.05 was considered to be statistically significant. Error bars in figures indicate SEM.

## Supplementary information

Supplemental figures
