# Supplementary material for: Orally desensitized mast cells form a regulatory network with Treg cells for the control of food allergy
Source: Mucosal Immunol. 2020 Dec 10;14(3):640–51. doi: 10.1038/s41385-020-00358-3 (PMC8075951; doi:10.1038/s41385-020-00358-3)
Supplement: Supplementary file 1 — Supplemental figures [file 41385_2020_358_MOESM1_ESM.pdf]

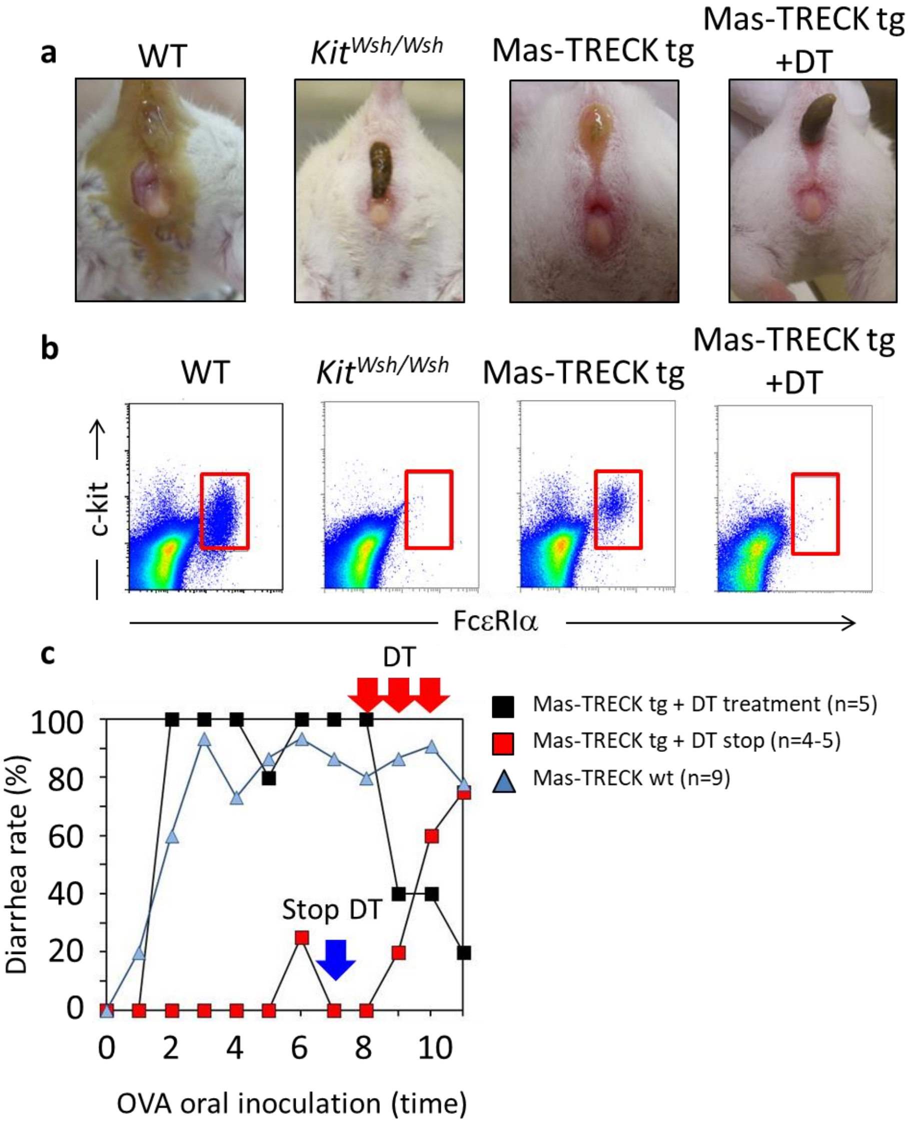

Supplemental Figure 1. Mast-cell (MC)-dependent allergic diarrhea

(a) An attempt was made to induce allergic diarrhea by ovalbumin (OVA) administration to wild-type (WT) and *Kit*$^{W\text{-}sh/W\text{-}sh}$ BALB/c mice and to Mas-TRECK transgenic (tg) mice with or without diphtheria toxin (DT) treatment. Representative feces are shown in the photos. (b) Dot plots of colonic mast cells are shown as c-kit^{+} FcεRIα^{+} cells. (c) Occurrences of allergic diarrhea in Mas-TRECK mice after oral administration of raw 50mg OVA. DT was given every day to other Mas-TRECK mice and then stopped on day 7; and not given until day 8 in a third group. Blue arrow indicates when DT administration was stopped, and red arrows indicate timing of administration of DT.

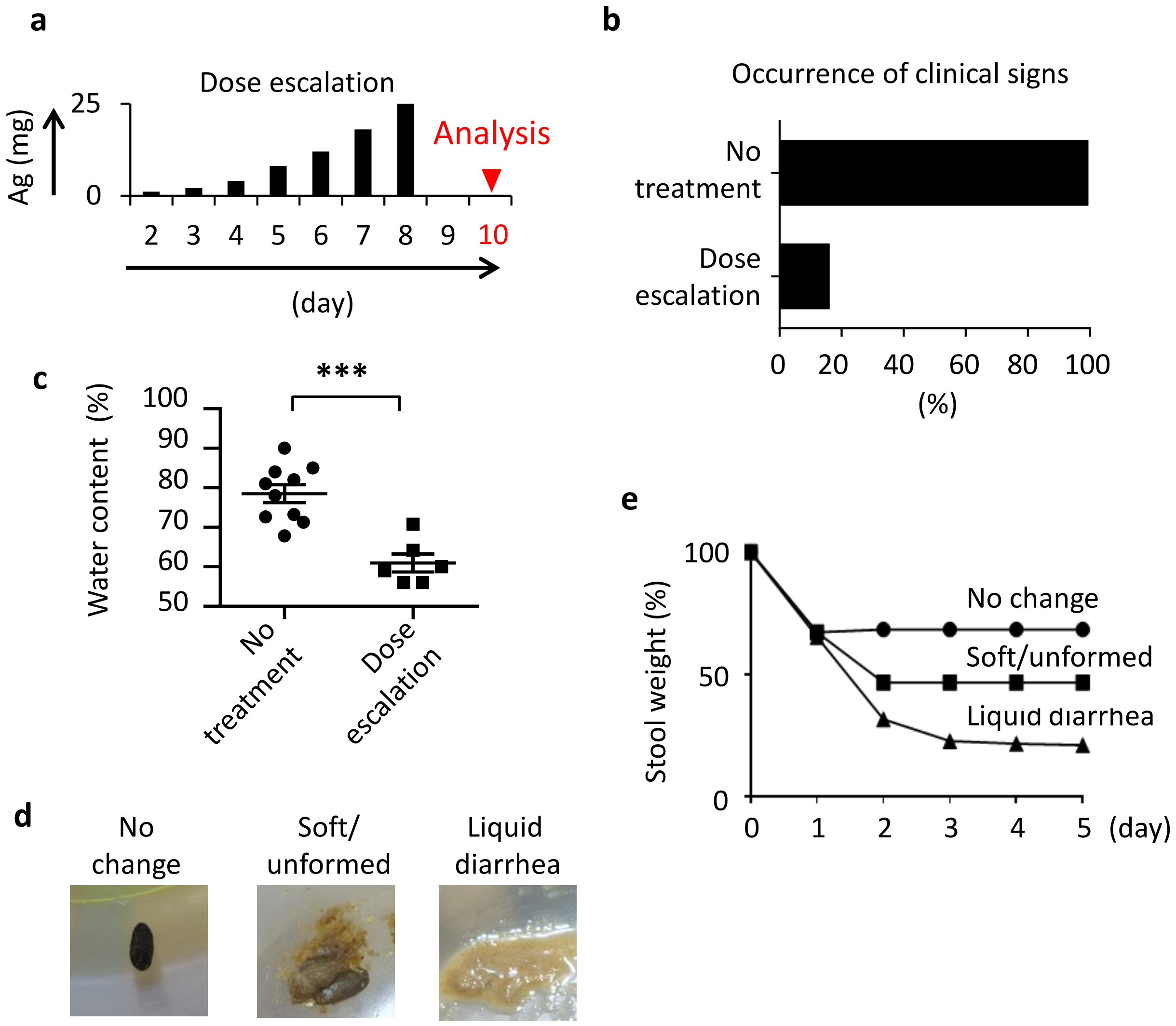

Supplemental Figure 2. Oral immunotherapy (OIT) protocols for treating allergic diarrhea in mice

(a) Protocol for dose escalation (up to 25 mg of heated OVA) is shown. “Ag” indicates antigen.
(b) Diarrhea occurrences in BALB/c mice. A group did not receive OIT indicates no treatment (c) The water content of the stool was measured; percentages are shown. *** P < 0.001. (d) Criteria used for judging stool appearance. (e) Stool dry weight as a percentage of total stool weight in the three stool-appearance groups.

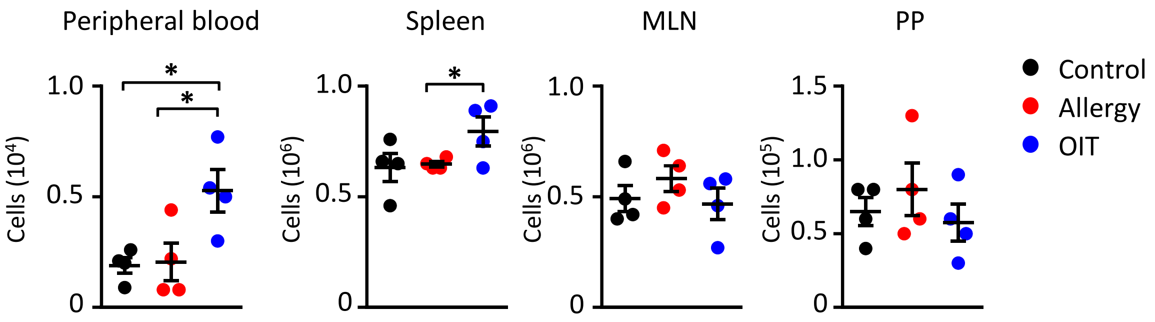

Supplemental Figure 3. Increase of Treg in the systemic compartment after OIT treatment

Treg cells (Foxp3^{+} CD25^{+} CD4^{+} CD3^{+}) were analyzed by flow cytometry. Cell numbers in the peripheral blood, spleen, mesenteric lymph nodes (MLN), and Peyer's patches (PP) are shown. n = 4; * $P < 0.05$.

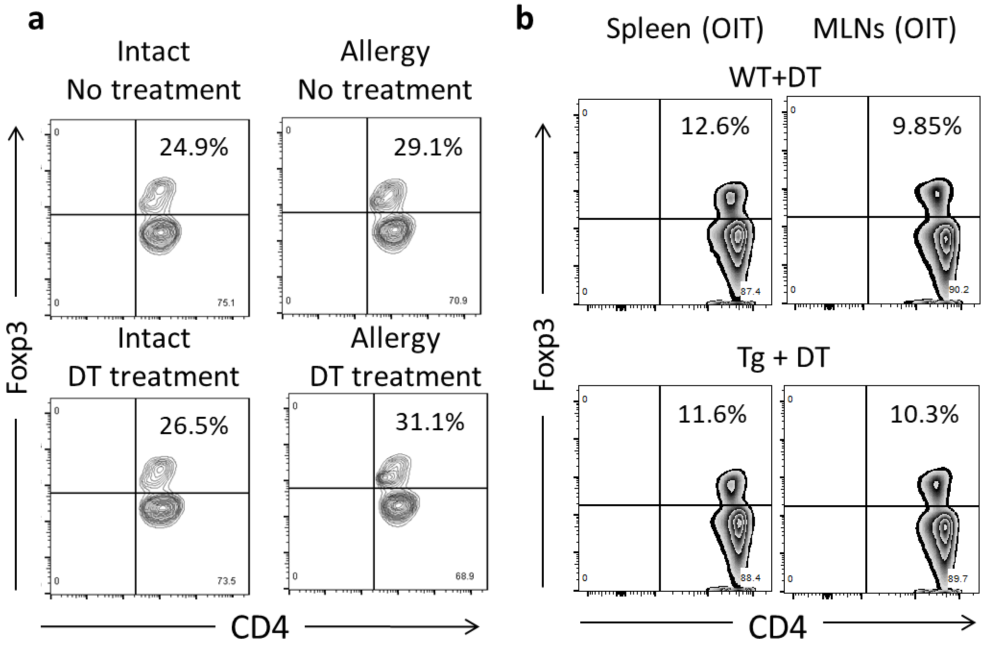

Supplemental Figure 4. Quantification of Tregs in the allergic state

(a) Colon Tregs of intact (WT) mice or allergic Mas-TRECK transgenic (tg) mice with or without diphtheria toxin (DT) administration were analyzed. Percentages of $Foxp3^+$ cells among $CD4^+$ T cells are shown. (b) Oral immunotherapy (OIT)-treated WT or Mas-TRECK tg mice given DT were sacrificed and Tregs in the spleen and mesenteric lymph nodes (MLNs) were analyzed by flow cytometry and cell sorting.

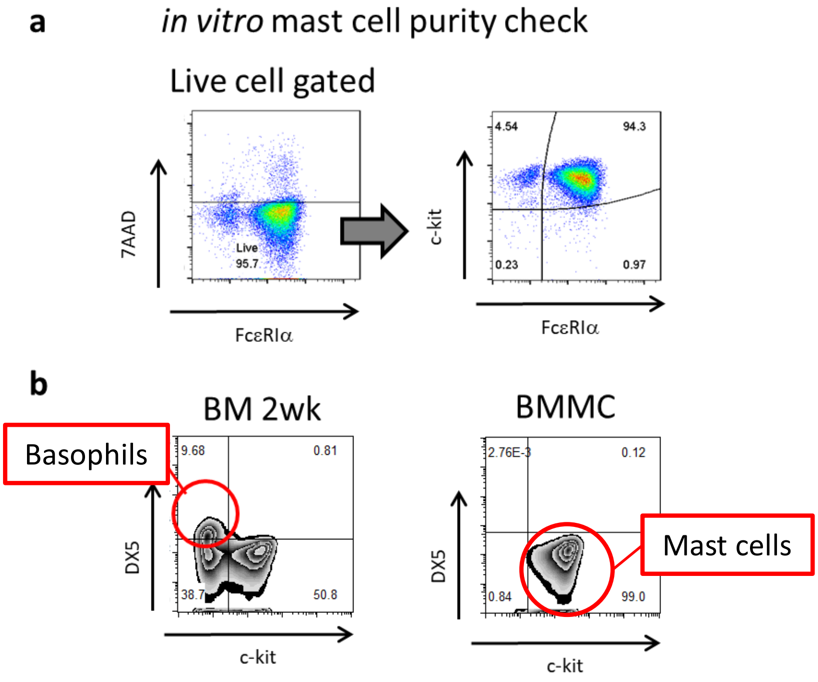

Supplemental Figure 5. ***in vitro*** **mast cell purity check**

(a) FACS plots of BMMC were shown. MC were derived from BALB/c mice with IL-3 contained medium for more than 6 wks. Represent data of purity of MC was shown. (b) no contamination of basophils (CD49b/DX5^{+} ckit^{neg} cells) were confirmed.

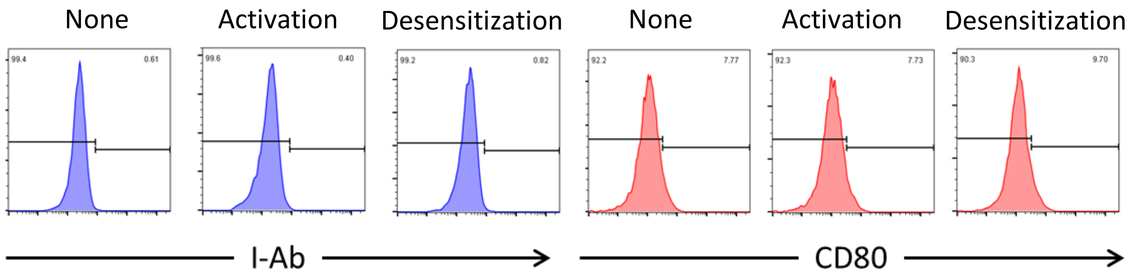

Supplemental Figure 6. No induction of antigen presentation in mast cells

Major histocompatibility complex class II (I-Ab) and CD80 expression was analyzed by flow cytometry and cell sorting. Data representative of three separate experiments are shown.

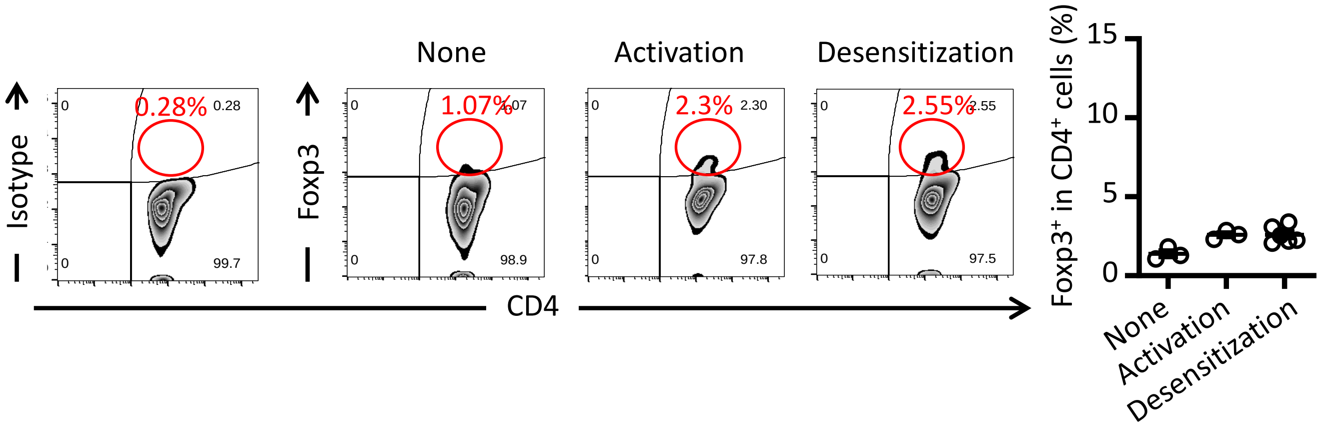

Supplemental Figure 7. Induction of Tregs from naïve CD4$^+$ T cells in MC cocultures

Desensitized bone marrow MCs were cocultured with naïve CD4$^+$ T cells from WT mice. Data are representative of three independent experiments for IgE-treated and activated MCs and of six independent experiments for desensitized MC cocultures (right panel).

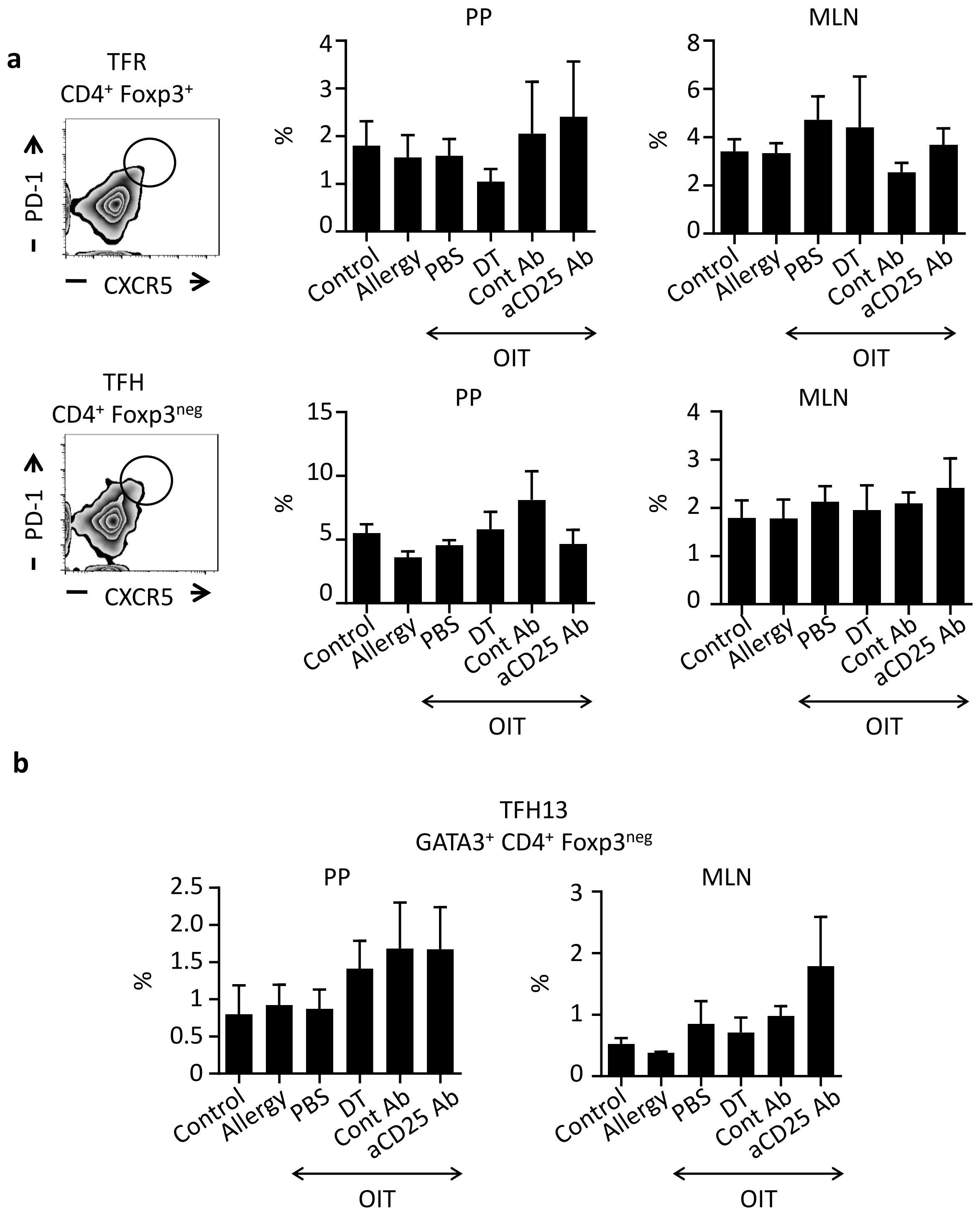

Supplemental Figure 8. Quantification of TFH and TFR cells during OIT

(a) TFH cells (Foxp3^{neg} PD-1^{+} CXCR5^{+} CD4^{+} CD3^{+}), TFR cells (Foxp3^{+} PD-1^{+} CXCR5^{+} CD4^{+} CD3^{+}) , and (b) TFH13 cells (Foxp3^{neg} GATA3^{+} PD-1^{+} CXCR5^{+} CD4^{+} CD3^{+}) in Peyer's patches (PP) and mesenteric lymph nodes (MLN) with and without OIT (see Figure 3a) were analyzed by flow cytometry. n = 4–6.

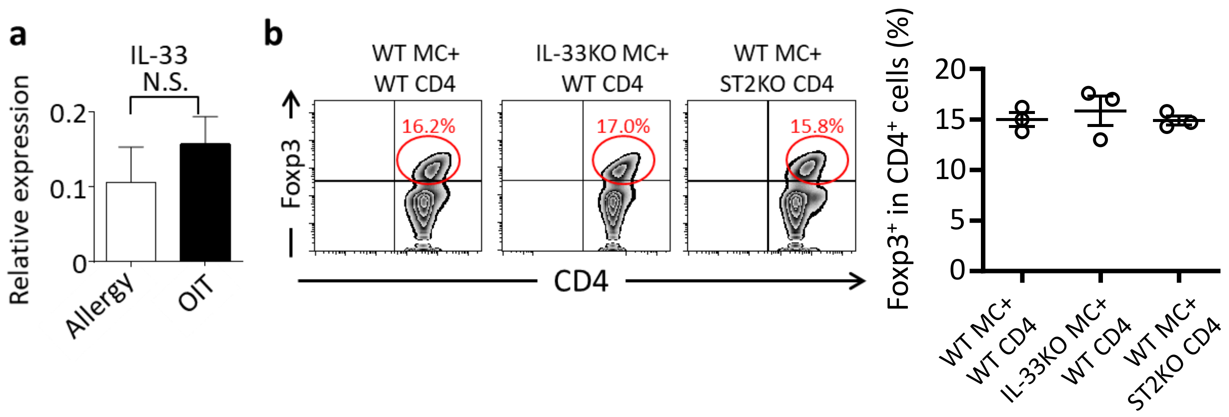

Supplemental Figure 9. Dispersive roles of IL-33 in desensitization-mediated Treg expansion

(a) Gene expression of *in vivo* sorted mast cells was analyzed by quantitative RT-PCR. Each result was normalized against the expression of *Gapdh.* N.S. indicates not significant. (b) Desensitized bone marrow MCs from wild-type (WT) or IL-33-deficient mice were cocultured with $CD4^+$ T cells from WT mice or mice deficient in ST2, the receptor for IL-33. Data are representative of n=3, shown in right panel.

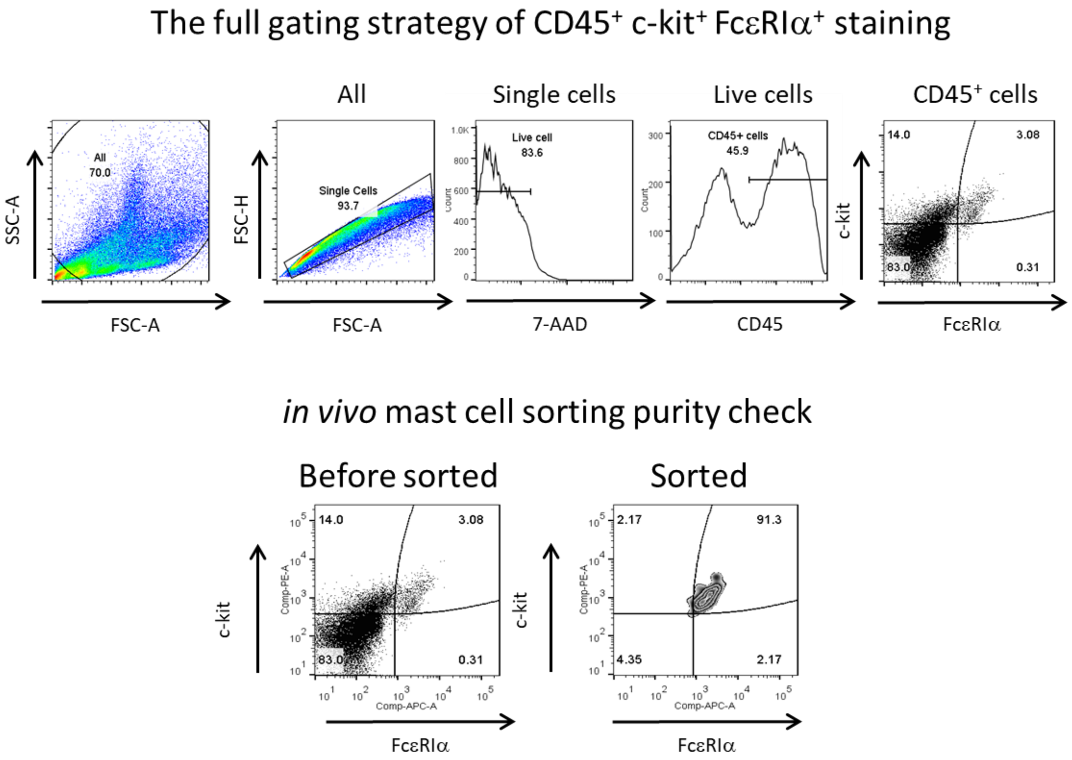

Supplemental Figure 10. The full gating strategy of CD45$^+$ c-kit$^+$ FcεRIα$^+$ staining

Lamina proprial cells were stained with CD45, c-kit, and FcεRIα and gating strategy of flow cytometry and representative of ***in*** *vivo* MC sorting purity were shown.

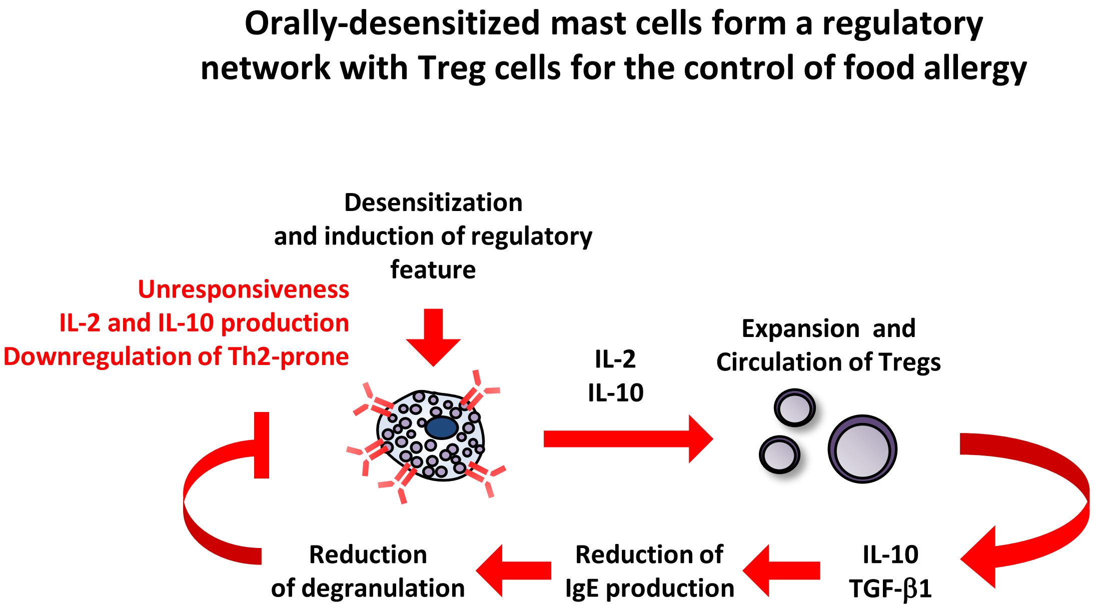

Supplemental Figure 11. Orally desensitized MCs form a regulatory network with Treg cells for the control of food allergy

Desensitized MCs produce IL-2 and IL-10 and induce a regulatory network via expansion of the Treg population, consequent reduction of IgE production, and degranulation of MCs. This regulatory network is required for successful OIT.
